# Benefits and challenges of upcoming microbial plant protection applications sustaining planetary health

**DOI:** 10.1016/j.isci.2025.113557

**Published:** 2025-09-15

**Authors:** Kalliope Κ. Papadopoulou, Antonis Chatzinotas, Belén Guijarro Diaz-Otero, Günter Brader, Dimitrios G. Karpouzas, Monica Garces Ruiz, José Luis Alonso Prados, Stéphane Declerck, Loukia M. Kellari, Angela Sessitsch

**Affiliations:** 1 University of Thessaly, Department of Biochemistry and Biotechnology, Larissa, Greece; 2 Helmholtz Centre for Environmental Research - UFZ, Department of Applied Microbial Ecology, Leipzig, Germany; 3 Institute of Biology, Leipzig University, Leipzig, Germany; 4 German Centre for Integrative Biodiversity Research (iDiv) Halle-Jena-Leipzig, Leipzig, Germany; 5 Consejo Superior De Investigaciones Científicas, Instituto Nacional de Investigación y Tecnología Agraria y Alimentaria, Madrid, Spain; 6 AIT Austrian Institute of Technology, Bioresources Unit, Tulln, Austria; 7 Université catholique de Louvain, Laboratory of Mycology, Earth and Life Institute, Louvain-la-Neuve, Belgium

**Keywords:** Biological sciences, Biotechnology, Plant biotechnology, Plant biology, Interaction of plants with organisms, Agricultural science

## Abstract

Plant disease outbreaks pose severe risks to global food security. Due to climate change, new diseases are expected to emerge, and the current use of chemical pesticides poses risks to environmental and human health. In the last decade, alternative plant protection agents of microbial origin have been developed, which also raise great expectations in the industry. Current products primarily represent individual microbial strains, either fungi or bacteria, which occasionally fail under field conditions due to various factors while their regulatory status differs globally. Recently, more diverse applications have started to emerge, ranging from microbial consortia, phages and protists to microbiome modulation or soil translocation. Integrated solutions, incorporating artificial intelligence are also proposed. In this review, we discuss the opportunities and challenges of these solutions, providing specific examples and discuss the regulatory needs for their market entry as well as their relevance for improving food security and planetary health.

## Graphical abstract

**Figure:**
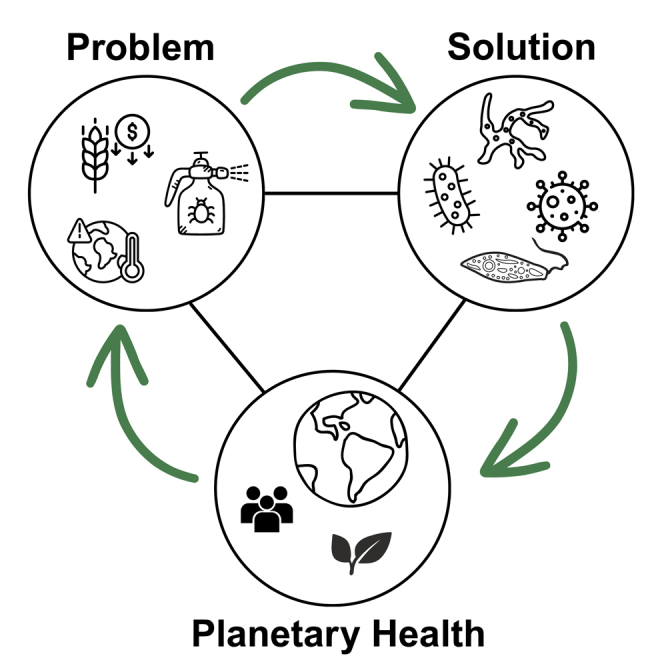

## Introduction

Plants provide about 80% of our food and are the economic basis for millions of farmers. They are the source of air we breathe and play a vital role in both environmental and human health. However, plant pests and diseases cause about 40% of annual crop losses, threatening global food security. Furthermore, climate change and intensification in agricultural and forest management practices may lead to more severe as well as emerging plant diseases. Farmers frequently use pesticides to secure high yields. Among them, chemical pesticides are still a cornerstone of modern agriculture, despite concerns about their widespread occurrence in natural water resources, soils and the food chain. It is characteristic that over 40% of the pollutants included in the watch list of water pollutant in Europe are chemical pesticides.^1^ Furthermore, pesticides have been associated with a decline of biodiversity, and several of them have been listed as potential endocrine disruptors and/or carcinogens.^2^ In addition, their frequent use in agricultural fields has resulted in reduced efficiency against pests and diseases due to the emergence of resistant fungal pathogens and insects or because of their degradation by soil-borne microorganisms.^3^^,^^4^ Worldwide, weeds have evolved resistance to 168 different herbicides, and the annual cost of herbicide resistance in England just against *Alopecurus myosuroides* (black-grass) is £0.4 bn.^5^^,^^6^

Pesticides contribute to climate change throughout their life cycle with the manufacture of one kilogram of pesticide requiring, on average, about 10 times more energy than one kilogram of nitrogen fertilizer.^7^ Furthermore, to give an example, it is now estimated that pesticide production and application in apple orchards and viticulture accounts for 51% and 37%, respectively, of total greenhouse gas emissions.^8^ Climate change will lead to high temperature and precipitation levels that will amplify the risk for crop infection, increase prevalence and prolonged developmental stages of soil-borne pathogens and insect pests.^9^ At the same time global food demand is anticipated to increase by 35%–56%.^10^ All these factors are expected to lead to an increasing demand for chemical control of pests and diseases, higher and more frequent application rates,^11^ a scenario that will amplify the deterioration of environmental quality and human health. To mitigate such undesirable effects different avenues are pursued, such as the development of disease-resistant crops, decision support tools that provide information to farmers on plant protection actions and the use of low-risk (biocontrol) pesticides, including microorganisms, plant extracts, semio-chemicals or dsRNA pesticides. The global biocontrol market is expected to rise from 6.5 billion USD in 2022 to 18.2 billion USD by the year 2029, with a double-digit % CAGR and microbial biocontrol agents have the greatest market share.^12^ Thus, the successful implementation of low-risk pesticides, in particular microbial biocontrol agents and upcoming microbial solutions, is likely to have a major impact on planetary health. However, the application of microbial solutions has been limited due to variable success in the field, which requires improved solutions as well as of unresolved regulatory issues.

## Current microbial applications and their limitations

The use of microbial biocontrol agents is still a strongly emerging market. Currently, most applications are based on single microbial strains acting against pests and plant pathogenic fungi; products based on *Bacillus thuringiensis* (Bt) but also strains of *Beauveria bassiana* and *Metarhizium* spp. are broadly used against insect pests.^13^ For the control of aerial and soilborne fungi, strains belonging to *Bacillus* (mainly *B*. *subtilis*, *B*. *velezensis* and related species) and *Trichoderma* spp. (mainly *T*. *afroharzianum*, *T*. *asperellum*, *T*. *atroviride*, *T*. *gamsii*, and *T*. *harzianum*) are the most widely used.^14^ Other genera or species, such as *Pseudomonas* or *Coniothyrium minitans*, have been explored, but are found in a comparably low number of products.^13^ In addition to bacterial and fungal applications, few products based on baculoviruses acting as enthomopathogens against chewing insects are on the market.^15^

An advantage of microbial biocontrol in comparison with chemicals is that resistance is less likely to be developed, unless microbial plant protection products are overused. For example, while Bt strains have a long history of effectiveness over the last four decades, only certain cases of resistance in pest populations have been observed.^16^ Despite such successful applications, the efficacy of first-generation microbial inoculants is often highly variable, depending on the conditions of the receiving environment (e.g., physico-chemical properties), plant genotype, competing native microbial communities, agricultural practices, inoculant formulation, and application method.^17^ A chief challenge in developing microbial products has been the lack of proper formulations conferring viability and stability of the biocontrol agent during storage and transport. Apart from requiring specific conditions to ensure shelf-life (e.g., storage under low temperature or the addition of protective agents such as UV stabilizers to reduce microbial degradation), the choice of formulation needs to match the application method (e.g., seed coating, spraying) to ensure effectiveness and depends on the microbial inoculant. Common formulation types include liquid forms like suspension concentrates and emulsifiable concentrates, as well as solid forms such as wettable powders, granules, and dusts. More advanced formulations include encapsulated products for controlled release and enhanced protection, and bait formulations that attract target pests. Other specialized forms include oil-based formulations, tablets for aquatic use, and water-dispersible granules.^18^

A common bottleneck is the poor establishment of microbial plant protection solutions in the soil or plant environment, substantially reducing efficacy under field conditions. Often, single-strain products may fail to compete with the natural soil and plant microbiota and show poor establishment, further diminishing their effectiveness.^19^ This is partly due to the fact, that we still have limited understanding of ecological interactions of the formulated inoculants within complex microbial communities. To improve field success, it is of key importance to select not only efficient biocontrol strains, but also to select strains, which are able to interact in a desired manner with resident communities and the environment. Concepts like niche theory, community assembly, and priority effects have been proposed—and in some cases experimentally explored—to explain the ecological failures of introduced microbial strains in the complex natural communities.^20^^,^^21^^,^^22^ Recent investigations have revealed shifts within soil microbial communities due to microbial inoculation, even if the inoculant strain did not well establish.^23^

In terms of efficacy, unlike broad-spectrum chemical pesticides, most single strain applications target specific pests or pathogens, which limits their broad applicability, while non-target effects remain a concern. In addition, bacterial biocontrol agents may carry antimicrobial resistance genes, and these genes could potentially be transferred to other microorganisms or pathogenic bacteria. To overcome these limitations, there is a need for a second generation of microbial plant protection applications to achieve reliable disease control without harming ecosystem functions and planetary health.

Several microbial mechanisms have been identified for combating plant diseases via direct or indirect mode of actions. Direct effects on the pathogen are mediated by the production of secreted compounds with biocidal activity. For example, lytic enzymes hydrolyzing chitin, proteins and cellulose are effective antimicrobial, antibiofilm, and sporicidal agents. Antimicrobial peptides disrupt cell membranes, causing cell death by osmotic shock and non-ribosomal lipopeptides (e.g., fengycin and surfactin), produced by many *Bacillus* spp., have strong antibacterial and antifungal activities.^24^^,^^25^ The production of allelochemicals, volatile organic compounds (VOCs) and quorum quenching (i.e., the disruption of the quorum sensing signals) have also frequently been reported as means of plant protection.^26^ Mycoparasitism describes the nutrient-depletion or fungicidal activity often observed in certain fungal-fungal interactions. Additional mode-of-actions of plant-protecting microorganisms involve the modification of the environment and nutrition of the plant.^27^ Indeed, improved plant nutrition is regarded as a first line of plant defense due to the direct involvement of mineral elements in plant protection. Competition and niche exclusion are also well-known mechanisms exerted by beneficial microorganisms, acting to limit/block access of nutrients to pathogens.^28^ Furthermore, induced resistance, which enables plants to increase their resilience against pests and pathogens by stimulating their own immunity, is an important mechanism that may be triggered by plant-associated microbiota.^29^

Upcoming microbial solutions will mostly show similar mechanisms as individual strains. However, synergistic interactions between microorganisms are anticipated to trigger multiple plant protection effects and may influence the environment differently. Generally, the complex interactions within microbiota and with their environment have been only poorly explored so far. Successful microbial interventions require an understanding of the complex and dynamic interactions within agricultural ecosystems and ecological theories support the use of diverse microbial consortia, integrated with other soil health practices. Indeed, due to the rising interest in applying microbial consortia, the number of studies taking higher complexity interactions into account is increasing, aiming to provide efficient solutions, tailored to specific environmental conditions.

## Microbial consortia

Considering the current limitations of microbial biocontrol agents, the industry has initiated more sophisticated approaches toward the development of successful microbial products for agriculture, which involve improved screening and selection to achieve strains that are adapted to the targeted (plant) conditions (e.g., via conditioning microorganisms to the plant environment). The microbial ecology under field conditions, besides biocontrol functions, is also increasingly considered in strain selection procedures. It can be expected that these efforts will lead to the identification of more efficient microbial biocontrol solutions.

Several upcoming biocontrol products make use of microbial consortia (frequently termed synthetic communities or SynComs). This advancement was triggered by recent research on the elucidation of microbial functions and interactions of microbial consortia members.^30^ Currently, these consortia typically contain only two to three microbial strains, which can be classified as low-complexity/diverse-taxonomy microbial consortia. Multiple approaches are used to design such consortia. One approach is to combine strains with synergistic activities, typically strains employing different mechanisms to control the pathogen or pest. For example, one strain produces a secondary metabolite against a certain pathogen, whereas another strain or other consortium members outcompete the pathogen or produce another metabolite.^31^ Furthermore, microbial strains that have similar functions (e.g., all strains outcompeting or antagonizing the pathogen) but perform well under different environmental conditions (e.g., showing different pH or temperature ranges) can be combined. Several studies have demonstrated the efficacy of microbial consortia that combine biocontrol fungi, primarily *Trichoderma* spp., with bacterial biocontrol strains.^32^ In another study, selected bacterial and fungal strains based on their antagonistic activities against root and foliar pathogens of tomato were established in various combinations, with the aim to extend the target pathogen range.^33^ Prigigallo et al. designed a consortium of *Trichoderma virens*, *Pseudomonas chlororaphis*, and *Bacillus velezensis*,^34^ which was applied in a non-simultaneous manner (to avoid cross-inhibition) and significantly reduced the incidence and severity of Fusarium wilt of banana.

A more elaborated approach is to design microbial consortia, informed by microbiome characteristics. The focus, in this case, is on core and keystone microbiome taxa. Core taxa are present across environments and can be also defined based on important functions. They seem to play a key role in organizing microbiome assembly,^35^ whereas keystone taxa are highly associated with other microbes in microbial networks.^36^ Designing microbial consortia based on microbiome assembly, core abundance and functions have high potential to produce microbial inoculants that establish better than those based on single strains. For example, this approach was used to design a microbial consortium consisting of core microorganisms isolated from grafted watermelon rhizosphere samples.^37^ These core strains protected the plants against *Fusarium oxysporum* via synergistic effects. Furthermore, the role of secreted low molecular weight molecules acting as chemical mediators in the functioning of microbial consortia needs to be considered.^38^

A compelling, ecology-based approach to use microbial consortia for plant protection was shown by Hu et al.^39^ The authors combined diverse, genetically related *Pseudomonas* strains to protect tomato plants from the bacterial pathogen *Ralstonia solanacearum*. In this approach the diversity effect was more important than strain identity. Similarly, Wang et al. applied bacteriophage consortia against the same pathogen.^40^ Again, diverse bacteriophage consortia increased plant protection in greenhouse and field experiments. We can classify these solutions as high-complexity/similar-taxonomy microbial consortia, requiring different safety considerations than other types of microbial consortia.

Soil translocation or soil transplantation, is a rather new approach that involves the shifting of whole microbial communities to restore specific functions and has been proven successful for the restoration of plant communities.^41^ However, this approach has not yet been used for plant protection purposes.

Despite the obvious advantages of microbial consortia, they also have limitations: they do not prevent competition with the local microbiota, their cost may be higher, especially if the organisms must be regulated and approved individually. The formulation and mode of application also need to be adapted. These are reasons why until now only a few products containing strain combinations have been registered for plant protection, with the first biocontrol product with a microbial consortium being registered in 2015.^27^

## Bacteriophages

Bacteriophages (i.e., viruses that infect bacteria) have received increasing attention as antibacterial agents in the production of agronomically important crops. Bacteriophage biopesticides are non-transducing and virulent, as temperate bacteriophages integrate into the host genome, confer resistance to superinfection and potentially transfer virulence factors.^42^ Conceptually, the most compelling argument for using bacteriophages to protect plants is their ability to grow rapidly within a host pathogenic bacterium and their high specificity, making them an ideal precision tool with potentially low or no side effects on other components of the soil/plant microbiome.^40^ Several studies have demonstrated in recent years the potential of bacteriophage-based biocontrol approaches for a few crops and plants.^43^ Different application strategies have been proposed for field conditions, depending on the pathogen lifestyle and infection strategy. These include post-harvest/storage control of infections,^44^ preventive treatment of seeds,^45^ or direct application to roots, flowers, plant wounds or the phyllosphere.^40^^,^^43^

A combination of different bacteriophages (i.e., a bacteriophage cocktail) has been found to be more efficient than a single phage at reducing pathogen density, disease incidence and disease index in greenhouse and field experiments.^40^ Multiple bacteriophages can target different receptors and have different infectivity profiles (even when genetically highly similar bacteriophages are combined),^40^ which together with the timing and sequence of exposure, is thought to limit the emergence of resistance.^43^ However, resistance development is a concern that needs to be addressed in more studies, as it may limit the application of bacteriophages for biocontrol. Most research on resistance development has been done in *in vitro*, however, the relevance in a significantly more complex natural setting is still unclear. A recent study showed that resistance development is context-dependent, cannot be studied *in vitro* alone and should therefore be investigated in ecologically relevant *in planta* setups.^46^ Furthermore, the potential of bacteriophage-mediated horizontal transfer of undesirable genes has to be considered in a risk assessment while the transfer of genes via generalized transduction is a major concern for temperate phages, some lytic phages have also been demonstrated to mediate generalized transduction.^47^

The low environmental stability of applied bacteriophages or consortia due to abiotic and biotic factors, such as high temperature, desiccation, and high solar irradiation may challenge environmental application. Low-cost solutions that increase bacteriophage survival may include applications together with an avirulent host carrier,^48^ formulations containing light-absorbing substances and proper timing of field application during the day.^49^^,^^50^ Bacteriophage-based biocontrol has great potential for farmers to reduce plant pathogens while increasing productivity and minimizing environmental impact. Although the market has seen the registration of the first commercial products, clear guidelines for the regulation processes are still needed.^51^

## Protists

Protists represent the majority of eukaryotic diversity and play an important role as phototrophs, saprotrophs and heterotrophs, as well as in symbiotic interactions. They are therefore important for the implementation of half of the UN Sustainable Development Goals.^52^^,^^53^ In soils, they are particularly critical because they are the dominant and often selective consumers of bacteria and fungi.^54^ Their effects on plant and soil microbiomes, growth and health suggest that protist-based solutions could provide a sustainable alternative to chemical treatments of microbial plant pathogens, but research in this area is still in its infancy.^55^ Fungal or bacterial pathogens were suppressed by direct predation by inoculated strains, while selective grazing on rhizosphere bacteria may favor the selection of beneficial bacteria with enhanced phytopathogen inhibition potential.^56^ Recent work has shown a correlation between bacterial anti-predation strategies and plant-beneficial traits, such as the biocontrol potential index (BPI),^57^ which should be further studied as an indirect mechanism in pathogen control. However, while the few published studies demonstrate considerable potential, they remain rather preliminary. More convincing scientific evidence on the potential of protists for targeted plant protection in large-scale studies is required. Also, there is a need for (1) new approaches for high-throughput strain isolation, (2) characterization of desired functions, traits and interactions with plants and microorganisms, (3) affordable large-scale cultivation, and (4) validation of protist applications under combined laboratory, greenhouse, and realistic field conditions.^55^

## Microbiome modulation

To make effective and lasting use of the beneficial effects of plant microbiota, the modulation and precision modification of the plant microbiome could be a future approach. Recent studies indicate that the application of microorganisms in agriculture have great potential,^13^^,^^17^ and a knowledge-based modulation of the microbiome has been even brought in context with a second green revolution.^58^ How can such a targeted microbiome modulation be addressed? In gut microbiome research, specific modulation is achieved by prebiotics, including specific bacterial taxa and food ingredients.^59^ Various modulators are potentially responsible for microbiome modulation, including chemicals and antibiotics, food ingredients, non-digestible fibers and uptake of microorganisms, bacteriophages, and whole microbial communities, such as in transplanted feces.^60^^,^^61^ However, the exact modulating mechanisms and the role of keystone species, and their wider effects on the organism needs to be investigated in more detail.^62^ Other important parameters to be considered include application methods, doses or frequencies of modulations. Generally, there are still several obstacles to be overcome and tackled for more specific approaches to modulating the microbiome.^60^

In agriculture, similar mode of actions can be envisaged for modulating the microbiome as for improving the gut microbiome. However, an important, additional parameter that needs to be addressed is the complexity of the targeted microbial community. For instance, the rhizosphere hosts highly complex microbiota, whereas the plant endosphere is a low complexity environment. Factors shaping the plant microbiome composition include climatic and edaphic components and the host plant itself.^13^ Plant genetics has also been suggested to modulate plant microbiomes.^63^ Attempts to engineer crop microbiomes, via host genetics with the help of so-called M-genes, are important avenues for a future microbiome-assisted agriculture.^64^ A specific mechanism, via which plant genotypes influence the microbiome in the rhizosphere, is achieved by the release of a mixture of specific compounds and many reports identify certain compounds (e.g., coumarins and terpenes) as novel specific players in plant-microbiome communication.^65^^,^^66^ The usage of specific plant- or microbial-derived compounds, such as bacterial signaling or biofilm modifiers shaping the microbiome, can be a promising approach.^13^ The use of synthetic biology approaches including targeted CRISPR/Cas (plant or microbial) genome modification is promising for a very specific design of microbial consortia or even *in situ* microbiome engineering. Moreover, the plant microbiome could be targeted and actively altered by bacteriophages,^51^ which can not only supress directly pathogens, such as *Ralstonia solanacearum*, but also enhance microbial diversity and abundance of certain taxa. The latter, in turn, further suppress disease.^67^ Management practices, like crop rotations, tilling and fertilization also modulate the microbiome, and precision farming approaches could be used in the future to precisely steer microbiome functions. Furthermore, genetic modification of plant microbiomes to achieve certain functions is envisioned.^68^ Overall, the application of microorganisms, either as single strains or consortia, modulating compounds, nano-materials or precision farming approaches has the potential to modulate soil and plant microbiomes in a way to suppress plant diseases.^13^^,^^69^

## Integrated solutions and digital, AI-based tools

The application of new technologies is bringing new opportunities for precision pest management. To date, the advancements of DNA and RNA next-generation sequencing have allowed for the discovery and characterization of microbial species that are not culturable in laboratory conditions. This technology has enabled the understanding of the community composition and function of microorganisms that live in association with plants in the rhizosphere. Next-generation sequencing can help predicting the performance of inoculated microorganisms for biocontrol purposes in their recipient environment as well as their interaction with the plant and local microbial communities. In addition, integrative system biology encompassing genomic and network-based approaches are used to dissect plant-microbe interactions dynamics and associate microbial taxa and functions with plant phenotypes under diverse environmental parameters.^70^ Also, sequencing and cultivation-independent tools are developed that aim to better investigate microbial community assembly and functions.^71^ However, there is a knowledge gap in understanding the underlying genetic and genomic features of antimicrobial or biocontrol activities under a specific biotic or abiotic context. To overcome this limitation, Biggs et al.*,*^72^ have developed a machine-learning workflow to identify genomic features associated with antifungal activity and leveraged that information to discover additional taxa with the desired activity. Similar efforts are underway in the industry to integrate genomic data with predictive modeling to accelerate product development, demonstrating the translational value of these approaches (e.g.,^73^^,^^74^^,^^75^^,^^76^).

Coupled with the use of modern sequencing techniques, the future of microbial biocontrol will rely on tailor-made solutions based on the information collected through novel monitoring techniques, which are crucial for optimal pest management. The use of digital monitoring provides a comprehensive overview of the crop status at a specific time point, enabling the visualization of plant stress in different areas of the field or greenhouse. Examples of plant health monitoring provide an opportunity to optimize biocontrol practices by estimating disease severity upon the application of a biocontrol agent through hyperspectral imaging or infrared thermography.^77^^,^^78^ In addition, new available tools using drones, satellite imagery, automated traps or autonomous robots can be considered as precision pest management delivering solutions to the hotspots where the pest has reached an economic threshold.^79^ The deployment of microbial biocontrol products within such integrated pest management schemes, where multiple parameters are assessed to propose optimal solutions, can serve as an alternative and/or supplementary and responsible approach in a sustainable agricultural landscape. A constant understanding of plant-microbial interactions in the agricultural setting will allow for of *in-situ* characterization of microbial performance under different contexts, such as the developmental stages of the crop or during a disease outbreak. For instance, preventive measures could be applied for predicting a disease outbreak by monitoring changes in the microbial communities and plant phytohormonal profiles. During a disease outbreak or pest infestation, the monitoring of plant stress signals, but also microbial cues such as the metabolites or volatile emissions in the soil, could provide an indication of the plant stress status and a characterization of the environment where the microbial biocontrol agent has been introduced. The vast amount of data collected through continuous monitoring could help developing predictive scenarios that will enable a highly specialized use of microbial biocontrol in an integrated pest management agricultural setting.

## Benefits, challenges, and regulatory issues regarding the implementation of new solutions

Microbial solutions, including microbial consortia, bacteriophages and protists, offer transformative potential in sustainable agriculture by addressing critical challenges in pest and disease management. Microbial consortia enhance agricultural productivity through synergistic effects, combining multiple strains, to broaden pathogen control and improve resilience to environmental variability. Products designed on the basis of ecological interactions within microbial communities will be better able to establish themselves and perform their desired functions effectively. Bacteriophages are highly specific, targeting only bacterial pathogens without affecting beneficial microbes. Protists contribute to plant health through multitrophic interactions, controlling pathogens, enhancing nutrient cycling, and promoting plant growth. Ultimately, precision farming and microbiome modulation offer avenues to minimize external input and to benefit from natural functions to maintain a healthy plant system.

Despite these advantages, there are also challenges to address. Bacteriophages, while precise, are limited by their narrow host range, which restricts their ability to target multiple pathogen populations within a single crop. Additionally, a critical evaluation of the potential of resistance development and horizontal gene transfer of problematic genes requires more *in planta* studies. The environmental stability poses an additional challenge, as factors such as UV light, temperature variations and desiccation can reduce their efficacy in field conditions. Intellectual property protection, regulations and guidelines for their application in agriculture either do not exist or differ across countries.^80^ This further complicates the adoption of bacteriophages,^81^ with concerns about their indirect effects on microbiomes and their potential to contribute to antimicrobial resistance requiring rigorous evaluation.^82^^,^^83^ Protists, though promising, remain underexplored in agricultural applications due to limited research and a lack of standardized protocols.^84^ Their interactions with plant and microbial communities can be complex and unpredictable, raising concerns about potential unintended effects. Microbial consortia, while offering significant benefits, face difficulties in maintaining stability and compatibility among the strains under diverse environmental conditions. Developing cost-effective and stable formulations for consortia requires advanced technological approaches, and intellectual property issues add complexity due to shared biological traits among microbial species.^85^

Regulatory frameworks also present significant barriers to the adoption of these microbial solutions. In Europe, stringent guidelines under the Plant Protection Products Regulation (EC) No. 1107/2009 demand comprehensive safety and efficacy evaluations, making the approval process lengthy and costly. In the USA, the Environmental Protection Agency imposes strict requirements on bacteriophages and microbial consortia, particularly concerning antimicrobial resistance and environmental impacts. Canada’s Pest Management Regulatory Agency also evaluates these products but faces challenges with the limited local data, which delays approval processes. Australia mandates extensive field trials, adding to costs and timelines, while regulations for protists and consortia remain underdeveloped. In Asia, regulatory approaches vary widely; China is rapidly adopting microbial biopesticides but deals with enforcement and quality control issues, whereas India requires improved efficacy validation. In Brazil, despite an expanding biopesticide market, the registration process remains slow, with limited provisions for novel solutions such as consortia or bacteriophages.

In summary, while bacteriophages, protists, and microbial consortia hold immense promise for sustainable agriculture, addressing challenges related to environmental stability, regulatory complexities, and formulation development are critical. Streamlined regulations, increased public and private funding of research, and enhanced public awareness will be pivotal in unlocking their full potential to promote global food security and planetary health.

## Conclusions—Benefits for planetary health

**Figure 1:**
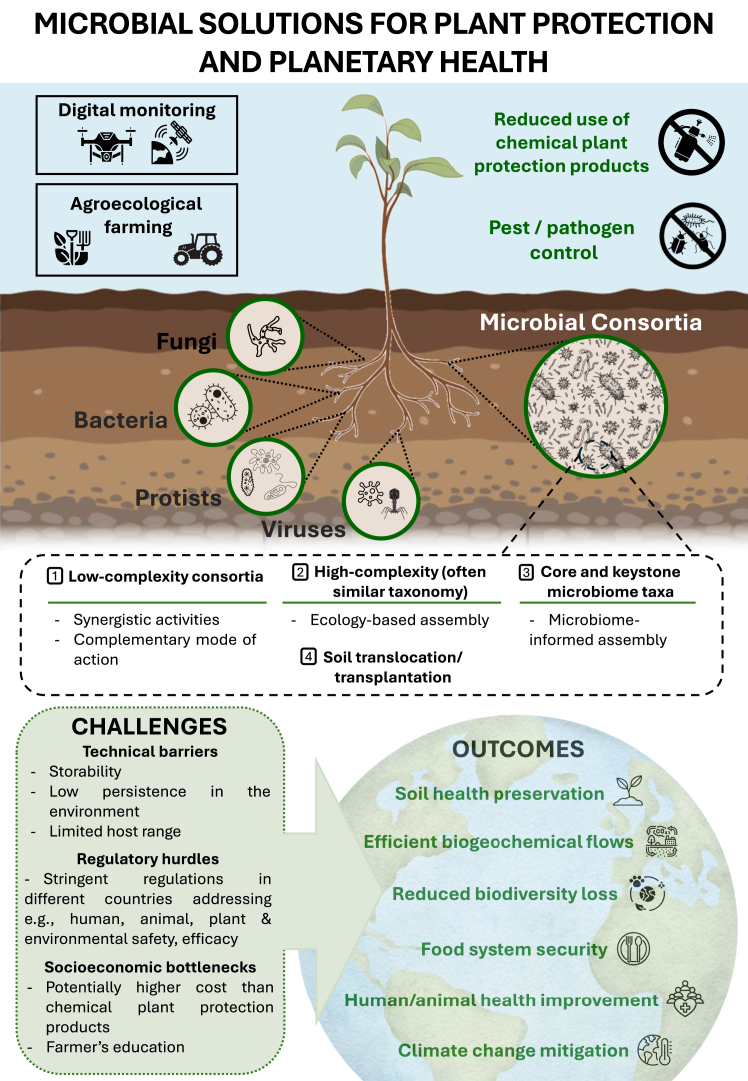
Microbial solutions for plant protection and planetary health

The global microbiome is universally recognized as essential to the functioning of planetary systems.^71^ With the omnipresence and interconnectedness of microbiomes across the biosphere and diverse range of activities,^72^ microorganisms are considered crucial drivers of planetary and ecosystem processes and, consequently, directly or indirectly associated with the different Sustainable Development Goals toward achieving a more sustainable future.^86^^,^^87^ Along these lines, the microbiome-based solutions and interventions outlined here, will enhance plant health and boost crop yields. They can also address post-harvest diseases, thereby supporting ecosystem services tied to sustainable food production and economic stability, improved food quality and safety and waste management. In addition, by replacing chemical pesticides, novel microbial inoculants and related biotechnological products are expected to contribute to restoring soil health, preserving ecological functioning and biodiversity, enhancing soil carbon sequestration and reducing greenhouse gas emissions. Thus, as climate change and land use practices continue to exert severe impacts on agricultural ecosystems, the deployment of microbial and microbiome-based products in a safe and coordinated way gains increasing attention as viable climate solutions (Figure 1).^88^ In addition, agricultural management like agro-ecological farming promotes soil health and a favorable soil/plant microbiome, which can be translated to reduced deterioration of biodiversity, improved nutrient cycling, mitigated antimicrobial resistance and reduced contaminants.^89^ Given the central role of healthy soils in planetary health, which encompasses human health and the well-being of the natural systems on which it depends,^75^ the integration of microbiome-based solutions into agriculture and plant protection strategies is deeply linked to advancing the objectives of the one health approach.^90^ This interwoven framework of developing novel biological and biotechnological solutions to tackle global challenges highlights the necessity of interdisciplinary collaboration, integrating environmental sustainability, agriculture, and public health to build a sustainable and adaptable future.

## Acknowledgments

Authors received funding by the Horizon Europe project RATION (“Risk assessment of low-risk pesticides”, HORIZON-CL6-2022-FARM2FORK-01, HORIZON-RIA, no. 101084163).

## Author contributions

Writing – original draft: K.K.P., A.C., B.G.D.-O., G.B., D.G.K., M.G.R., J.L.A.P., S.D., L.M.K., and A.S.; writing – review and editing: K.K.P. and A.S.; visualization: L.M.K.; conceptualization: A.S. All authors contributed to manuscript revision and approved the submitted version.

## Declaration of interests

The authors declare that they have no conflict of interest.
